# A systems approach towards remote health-monitoring in older adults: Introducing a zero-interaction digital exhaust

**DOI:** 10.1038/s41746-022-00657-y

**Published:** 2022-08-16

**Authors:** Narayan Schütz, Samuel E. J. Knobel, Angela Botros, Michael Single, Bruno Pais, Valérie Santschi, Daniel Gatica-Perez, Philipp Buluschek, Prabitha Urwyler, Stephan M. Gerber, René M. Müri, Urs P. Mosimann, Hugo Saner, Tobias Nef

**Affiliations:** 1 ARTORG Center for Biomedical Engineering Research, University of Bern, Bern, Switzerland; 2 LaSource School of Nursing Sciences, HES-SO University of Applied Sciences and Arts Western Switzerland, Lausanne, Switzerland; 3 Idiap Research Institute, Martigny, Switzerland; 4 School of Engineering, Ecole Polytechnique Fédérale de Lausanne (EPFL), Lausanne, Switzerland; 5 DomoHealth SA, Lausanne, Switzerland; 6 Department of Neurology, Inselspital, Bern, Switzerland; 7 Institute of Social and Preventive Medicine, University of Bern, Bern, Switzerland

**Keywords:** Geriatrics, Biomarkers

## Abstract

Using connected sensing devices to remotely monitor health is a promising way to help transition healthcare from a rather reactive to a more precision medicine oriented proactive approach, which could be particularly relevant in the face of rapid population ageing and the challenges it poses to healthcare systems. Sensor derived digital measures of health, such as digital biomarkers or digital clinical outcome assessments, may be used to monitor health status or the risk of adverse events like falls. Current research around such digital measures has largely focused on exploring the use of few individual measures obtained through mobile devices. However, especially for long-term applications in older adults, this choice of technology may not be ideal and could further add to the digital divide. Moreover, large-scale systems biology approaches, like genomics, have already proven beneficial in precision medicine, making it plausible that the same could also hold for remote-health monitoring. In this context, we introduce and describe a zero-interaction digital exhaust: a set of 1268 digital measures that cover large parts of a person’s activity, behavior and physiology. Making this approach more inclusive of older adults, we base this set entirely on contactless, zero-interaction sensing technologies. Applying the resulting digital exhaust to real-world data, we then demonstrate the possibility to create multiple ageing relevant digital clinical outcome assessments. Paired with modern machine learning, we find these assessments to be surprisingly powerful and often on-par with mobile approaches. Lastly, we highlight the possibility to discover novel digital biomarkers based on this large-scale approach.

## Introduction

Rapid population ageing is a global phenomenon that affects virtually every country on earth^1^. A major challenge in this regard is the increased disease burden and related disability associated with ageing, which is posed to further increase already high healthcare expenditures and significantly reduces older adults’ quality of life^1^. While there is unlikely to be a single solution to those challenges, concepts from precision medicine could play an integral part^2,3^. One idea here is the systems medicine approach, aiming to move from reactive to predictive, preventive, personalized, and participatory (P4) medicine^4,5^, a concept that was initially introduced by Leroy Hood and colleagues, more than two decades ago. A key aspect of systems, or P4, medicine is to monitor a holistic view of a person’s wellbeing in order to better manage their health and gain a deeper understanding of disease processes^4,6^. This is ought to be achieved by comprehensive systems approaches, like multi-omics profiling but more recently also by means of remote health-monitoring^3,6–8^. That is, by using connected sensing devices, such as smartphones, wearables or embedded internet of things sensing units, to continuously and objectively monitor health relevant information in everyday life^9,10^. This is in contrast to the currently often employed on-site visits that tend to merely provide a, potentially biased, snapshot of health states^3,11^. Today, such remote health-monitoring approaches are mostly focusing on a select few individual aspects of older adults’ lives, instead of employing more comprehensive systems approaches as advocated by P4 medicine. This focus, likely results in many phenotypes of health and disease to be missed and may limit the potential of large scale machine learning (ML) approaches. Furthermore, the used technologies tend to include sensing devices that are rather optimized towards younger demographics and may thus prove suboptimal for use by many (but certainly not all) older adults, particularly long-term. Something that could further increase the digital divide, potentially excluding seniors that would benefit the most from remote health-monitoring approaches. More comprehensive approaches towards remote health-monitoring in older adults, with a particular emphasis on ageing-inclusive sensing technologies, may thus be of high relevance for the future of remote health-monitoring in this growing demographic.

In the past decade an impressive number of studies have demonstrated the potential of digital measures, such as their use as digital analogs to biomarkers^12–17^ (hence, digital biomarkers^18^) and clinical outcome assessments (COAs)^19–22^ (hence, digital COAs^18^). Much of this research revolved around clinical research in neuropsychiatric disorders^23–25^ which are often linked to ageing and act as major contributors to disease burden^26^. In view of the above, it is not far fetched to assume that long-term monitoring of digital measures may provide continuous and objective information about an older individual’s functional status and health changes. This, in turn, could facilitate earlier and more personalised interventions^2,3^. Eventually, this may also help facilitate older adult’s independence, allowing them to stay at home longer, and increase their quality of life^9^. For instance, Rantz et al. show how sensor technologies linked to early alert systems led to better health outcomes amongst older adults^9^. Another example of long-term monitoring is presented by Austin et al., where they managed to assess loneliness using connected sensing technologies^27^. Finally, in one of the earliest long-term monitoring efforts related to older adults, Hayes et al. demonstrate that variations in sensor-derived gait speed and physical activity differ significantly between older adults with mild cognitive impairment (MCI) and healthy older adults^28^.

A large part of conducted research around digital measures, however, has focused on shorter-term studies in combination with mobile technologies, such as smartphones, smartwatches, as well as activity and fitness trackers. While there is no doubt that mobile technologies are a great way to derive health relevant information, they may not necessarily be ideal for long-term monitoring in the broader population of older adults. There are multiple reasons for this: (1) older adults tend to be more wary of novel technologies^29^; (2) since monitoring durations may become very long or even unlimited, it is ideal if there is no interaction (zero-interaction) with the system, as there is, for instance, evidence of wear-time-dependent compliance issues^30^; (3) there is a certain stigma attached to the use of wearable devices, whereby many older adults tend to fear being seen as frail if they wear a device - even if it is just an alarm clock^29^; (4) for seniors with potential memory issues, wearing and maintaining devices may not be feasible. As a result, many older adults that are affected by the digital divide^31^ may be excluded, this may be even more problematic in those of lower socio-economic background^32–34^, those living in harder to access rural areas^32^, or those living with certain conditions like cognitive impairments or late-life depression^33^. Not too surprisingly, most successful real-world, long-term research using sensor technology with older adults has focused on contactless, zero-interaction approaches^9,13,28,35–40^. Such technologies include passive infrared (PIR) motion sensors that capture an individual’s activity in a given room^9,28,35,36,38,41,42^, contact door sensors that can signal when a person leaves or enters the home^36–38,43^, pressure sensors on or under a mattress that capture sleep measures^9,36,39^, and electronic pillboxes to track medication adherence^44^, along with more obtrusive depth-sensing cameras that track silhouettes to detect falls and monitor gait parameters^9^.

Currently, digital measures are commonly used individually or by combining several specific ones based on concepts of interest. While this approach is entirely reasonable, it may limit the potential of digital measures as many potential characteristics of health and disease may simply go unnoticed. Furthermore, it is oftentimes not clear in advance, which, amongst correlated measures, may be the most relevant^13^. We therefore hypothesise that a more holistic systems approach — inspired by systems biology and applied to remote health-monitoring — may be highly promising. This involves deriving larger sets of digital measures, potentially in the hundreds to thousands, which may be particularly helpful in exploratory research and could also enable the creation of strong digital COAs by leveraging large-scale machine learning approaches^20^. This is in some ways analogous to more classical biological settings, where measurements can assess individual blood tests or genes (for instance, by means of single-nucleotide polymorphisms) but also whole sets, such as metabolomes or genomes, to identify new phenotypes of health and disease, such as is being proposed with the systems-oriented P4 medicine. In the context of zero-interaction, contactless technologies, a systems approach could also help to counteract some of the downsides of contactless technologies, such as lower accuracy as a result of the indirect measurement modality. In the context of digital measures, an extensive set of measures may be referred to as some sort of digital “ome”, such as a digital behaviorome^45^, or a digital exhaust^46^ — where the basic measurement unit is a digital measure. In this work, we will use the latter term as it is likely less controversial^47^.

Two notable real-world examples of using extensive sets of digital measures are studies by Cook et al.^42^ and Chen et al.^20^, which demonstrate the feasibility of using a digital exhaust based on wearable and contactless sensors to predict multiple clinical scores (in the former) and MCI (in the latter). Building on their work, we aim to evaluate the potential of a systems oriented approach towards long-term remote health-monitoring in the demographics of older adults. To this end, we first introduce an extensive set of 1268 well-documented digital measures that are entirely obtainable with sensing technologies that demonstrate extensive long-term, real-world evidence in older adults. Thus, all of these measures are based on a small set of zero-interaction, contactless, and cost-effective sensors that, as shown by Baettie et al., scale well to large ageing-related remote-monitoring projects^36^; this also means that these sensors should be compatible with most long-term monitoring projects in community-dwelling older adults. Using the resulting comprehensive set of digital measures, or digital exhaust, we further demonstrate how powerful ML based ageing-relevant digital COAs for fall risk, frailty, late-life depression, and MCI can be created. Finally, we highlight the possibility to leverage a digital exhaust to discover new potential digital biomarkers, demonstrating how a comprehensive systems approach could also help in establishing new phenotypes of health and disease.

## Results

### A zero-interaction digital exhaust

**Fig. 1:**
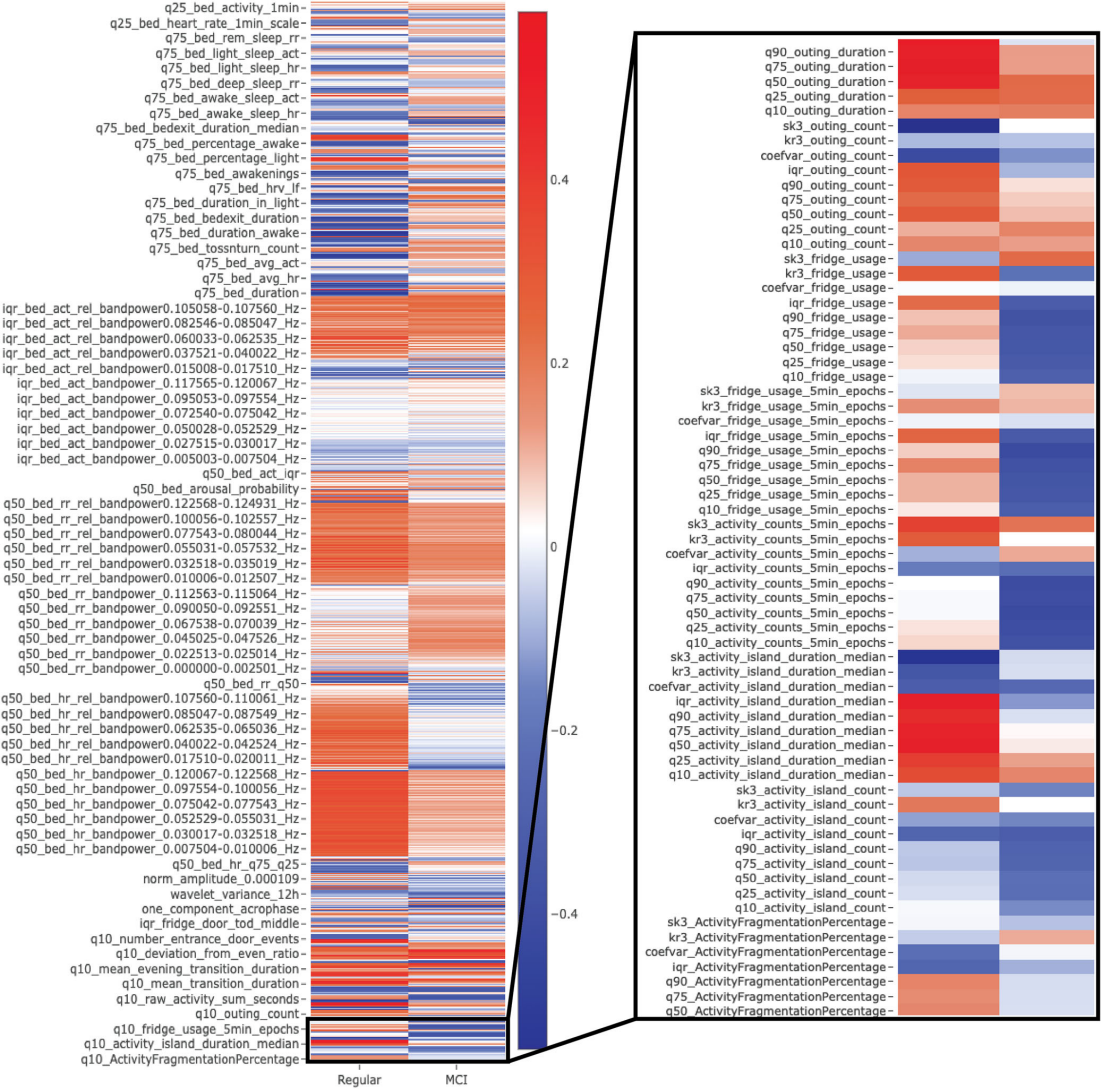
Exemplary visualizations of averaged digital exhausts. Depicts an example of z-normalised, averaged digital exhausts of participants with mild cognitive impairment (MCI) (based on a Montreal Cognitive Assessment screening < 23 points). Digital measures > 0 (in blue) indicate above-average values for that group, while < 0 (red) indicates below-average values. Many digital measures visually differ in both examples. It should be noted that this is a down-scaled visualisation, as not all measures would fit in the figure. For the complete and interactive version, see the supplementary online version (Note the zoom-in functionality).

We introduce a set of 94 hypothesis-driven base measures, from which we further derive a total of 1268 digital measures using aggregation and frequency analysis. All measures are obtainable through zero-interaction and privacy-preserving (neither video nor audio) contactless (thus no direct physical contact) sensing devices, which do not require any user interaction. Of these 1268 digital measures, 224 were extracted by means of PIR motion sensors in essential rooms (the entrance, bathroom, living room, bedroom, and kitchen) and magnetic door sensors on the refrigerator and entrance door. An additional 1044 measures were extracted on the basis of sleep data from a quasi-piezoelectric bed sensor placed under the mattress. Detailed descriptions and derivations, as well as associated hypotheses, are provided in the Supplementary Methods, together with a high-level overview of all presented digital measures (see Dataset 1). Furthermore, an extensive online version with interactive visualisations, along with additional data, including measure distributions and correlations with various ageing-relevant health indicators and outcomes, is available on GitHub ((https://narayanschuetz.github.io/digital-exhaust/) and serves as an online supplementary to this article. An example of averaged digital exhausts is shown in Fig. 1.

### Machine learning based digital clinical outcome assessments

Here, we demonstrate how the introduced digital exhaust could be useful for ageing and ageing related research. To this end, we created machine learning-derived digital COAs, aimed at automatically classifying ageing-relevant health outcomes. We created five datasets, one based on each clinical assessment, including fall risk, frailty, late-life depression, and MCI. This analysis is based on remote-monitoring data from two observational longitudinal pilot studies in Switzerland, where independently living, community-dwelling older adults were equipped with pervasive computing systems and monitored over the course of a year, while simultaneously being subject to regular visits and clinical assessments. The results on predicting ageing-relevant positive and negative health outcomes are summarised in Tables 1 and 2. The differences between using the digital exhaust alone versus using the exhaust in addition to demographics were minimal and, judging by overlapping 95% CIs, non-significant. The highest discriminative power, in terms of ROC AUC (area under the receiver-operating characteristic curve), was achieved with the Tinetti Performance-Oriented Mobility Assessment (POMA)-based fall-risk dataset when including both demographic and digital exhaust information (ROC AUC = 0.805). Notably, however, demographic information alone was sufficient to produce good performance (ROC AUC = 0.777) in this particular case. Performances on the fall-risk related Timed Up and Go Test (TUG), the MCI-related Montreal Cognitive Assessment (MoCA), and the frailty-related Edmonton Frail Scale (EFS) datasets were also relatively high, with ROC AUC values of 0.786, 0.780, and 0.704, respectively, when using only the digital exhaust. The worst performance was achieved with the dataset based on the Geriatric Depression Scale (GDS) (ROC AUC = 0.620), when using only the digital exhaust. Here, the difference between using only demographics versus using the digital exhaust was also minimal, with a slight but non-significant advantage in favour of the exhaust-only scenario. Overall, though, the addition of the digital exhaust resulted, in all cases, in higher ROC AUC and PrAUC (Area Under the Precision-Recall Curve) values than those obtained when using only demographic information. These differences were statistically significant in all but the POMA and GDS datasets (which were based on rather conservative, non-overlapping CI intervals). The largest differences were found with the MoCA and TUG datasets, for which the objectives were to identify participants with an indication of MCI or increased fall risk, respectively.

**Table 1:** Performance Evaluation based on ROC AUC.

Dataset	Demographics	Digital Exhaust	Digital Exhaust + Demographics
TUG	0.662 ± 0.041	0.786 ± 0.037	0.783 ± 0.036
POMA	0.777 ± 0.028	0.782 ± 0.035	0.805 ± 0.027
EFS	0.564 ± 0.034	0.704 ± 0.039	0.698 ± 0.038
GDS	0.602 ± 0.046	0.620 ± 0.048	0.616 ± 0.048
MoCA	0.430 ± 0.038	0.780 ± 0.039	0.757 ± 0.038

ROC AUC based results for the creation of machine learning-derived digital COAs based on clinical assessments for fall risk (TUG & POMA), frailty (EFS), late-life depression (GDS), and mild cognitive impairment (MoCA).

*TUG* Timed Up and Go Test

*POMA* Performance-Oriented Mobility Assessment

*EFS* Edmonton Frail Scale

*MoCA* Montreal Cognitive Assessment

**Table 2:** Performance Evaluation based on PrAUC.

Dataset	Demographics	Digital Exhaust	Digital Exhaust + Demographics
TUG	0.681 ± 0.035	0.816 ± 0.033	0.805 ± 0.034
POMA	0.615 ± 0.046	0.650 ± 0.047	0.678 ± 0.044
EFS	0.523 ± 0.041	0.625 ± 0.045	0.603 ± 0.044
GDS	0.397 ± 0.051	0.477 ± 0.054	0.458 ± 0.053
MoCA	0.653 ± 0.027	0.863 ± 0.028	0.839 ± 0.028

PrAUC based results for the creation of machine learning-derived digital COAs based on clinical assessments for fall risk (TUG & POMA), frailty (EFS), late-life depression (GDS), and mild cognitive impairment (MoCA).

*TUG* Timed Up and Go Test

*POMA* Performance-Oriented Mobility Assessment

*EFS* Edmonton Frail Scale

*MoCA* Montreal Cognitive Assessment

### Discovering novel digital biomarkers

**Fig. 2:**
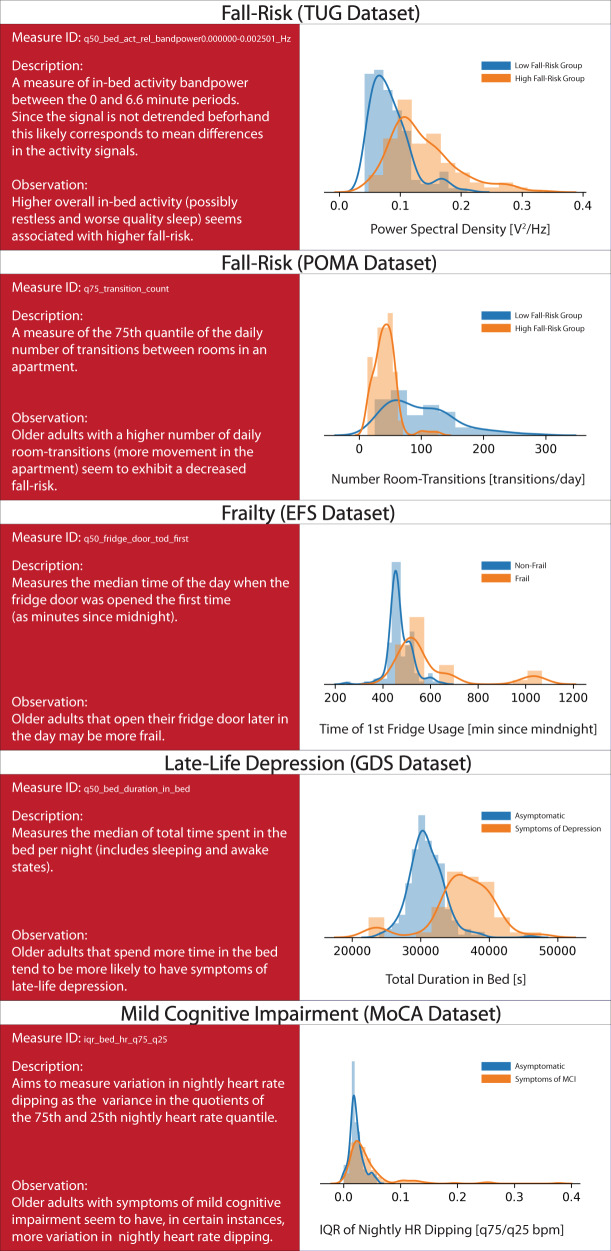
Most important digital measure for each outcome. Displays descriptions and density plots of the most important digital measure for each outcome. Across all density plots, blue indicates a positive/neutral outcome, while orange indicates a negative outcome. It should be noted that the proposed associations reflect correlation and not causation and should be interpreted accordingly.

**Fig. 3:**
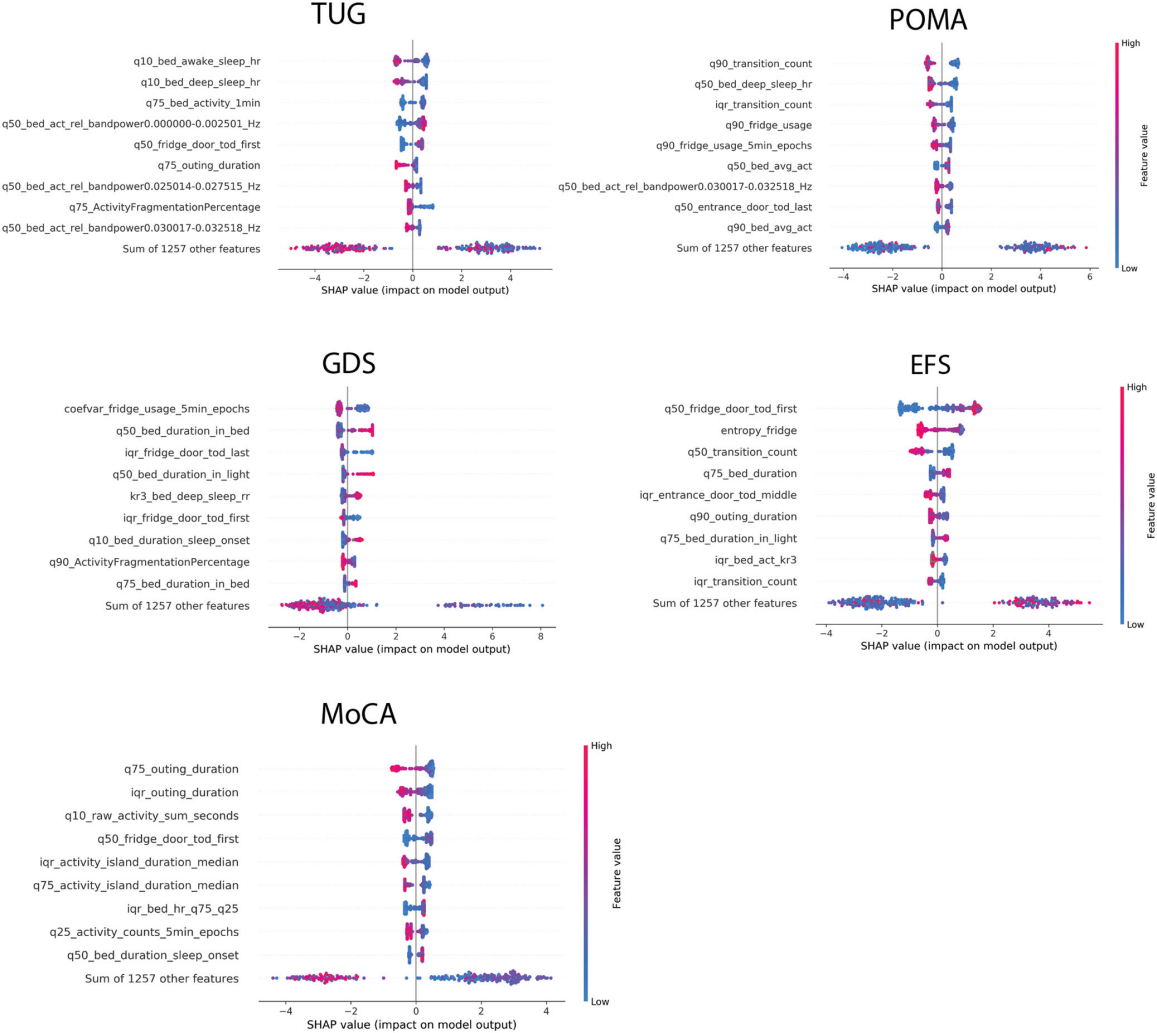
Beeswarm plot indicating digital measure importances across outcomes. Shows *beeswarm* plots of the 9 most important digital measures based on SHAP values on all outcome datasets: TUG (Timed Up and Go) *&* POMA (Performance Oriented Mobility Assessment = fall risk, GDS (Geriatric Depression Scale) = late-life depression, EFS (Edmonton Frail Scale) = frailty, MoCA (Montreal Cognitive Assessment) = mild cognitive impairment. Finally, the contributions of the sum of the remaining measures is displayed. Digital measures are ordered according to their importance, from top to bottom. The x-axis represents log odds, where values above zero indicate relevance for a negative outcome. Colouring further shows the direction of this association, where blue indicates lower values of a given measure and red indicates higher respective measure values. Detailed explanations of the individual measure names are given in the supplementary material or on the supplementary website. Note that these plots are based on models trained on the whole respective dataset and are therefore slightly different from the global importances shown in Supplementary Table 3, which are based on 100 simulation iterations.

The importance of individual digital measures (that may be interesting as digital biomarker candidates) with respect to the COAs were evaluated by means of SHapley Additive exPlanations (SHAP) values^48,49^. In Fig. 2, we present the most important digital measure, based on global SHAP values, across all 100 simulations of each single dataset - corresponding to the different COAs. A more detailed table, highlighting the top 10 highest-ranked measures based on global SHAP values, is available in Supplementary Table 3. Furthermore, in Fig. 3, we display *beeswarm* plots of SHAP values for the individual COAs. These show how the nine most important digital measures - as well as the sum of all other measures combined - influence the log odds ratio of having a negative health outcome on the various datasets. Values shown in Fig. 3 mostly align with the global SHAP importance rankings, although there are minor differences. Since the global SHAP rankings are based on 100 iterations (and thus 100 models), and since the importances shown in Fig. 3 are based on a single model, we generally place greater emphasis on the global SHAP values. Nonetheless, the *beeswarm* plots are still useful, as they provide insights into the direction of effects.

## Discussion

The present study evaluated the idea of a comprehensive digital exhaust for long-term remote monitoring in older adults. To this end, we introduced 1268 well-documented digital measures that aim to cover a large part of a person’s activity, behavior and physiology (extensive online documentation can be found on https://narayanschuetz.github.io/digital-exhaust/). Since most successful long-term remote-monitoring projects in older adults have employed zero-interaction, contactless sensing technologies, we based all introduced digital measures on such technologies. Using the resulting digital exhaust, in combination with real-world data, we could successfully create large-scale ML derived digital COAs for common ageing-relevant outcomes, including fall risk, frailty, MCI, and to a lesser degree late-life depression. Furthermore, we were able to showcase the discovery of potentially interesting new digital biomarkers related to the created digital COAs.

Beginning with the digital COAs, we found good discriminative performances across all but late-life depression, resulting in ROC AUC values of ≥ 0.7. Notably, these results are based on a very limited sample size, which makes it probable that this is a rather conservative estimate of what could be possible. In all cases, digital COAs based on the digital exhaust led to higher ROC AUC (see Table 1) and PrAUC (see Table 2) values than those obtained from only using demographics information. These differences were significant in three (TUG, EFS, and MoCA) out of the five outcome datasets, indicating that a digital exhaust captures information beyond just simple demographics. It is also notable that adding demographic information to the digital exhaust did not result in significantly better performance across outcomes, which may indicate that this type of information was already latently captured by the measures making up the exhaust. While we used digital COAs here more as an example for feasibility purposes, those could in fact be highly useful for continuously assessing an older adult’s health and functional status with respect to specific outcomes and may allow for the implementation of early preventive interventions, fitting in well with the proactive nature of precision medicine. For instance, if an older adult exhibits increased fall risk, it may be reasonable for them to see a fall prevention specialist, as opposed to taking action after a fall has already occurred.

Putting the above mentioned results into perspective, passive sensor-based fall-risk assessments were shown to yield AUC values in the range of 0.65–0.89^50^. It should be noted that these values were obtained using wearables by means of accelerometry and predominantly with very few digital measures of gait (and sometimes with accelerometer signal characteristics). Furthermore, none of the studies mentioned in this paper used long-term data, and some were performed under laboratory conditions far removed from real life. Our results, with ROC AUC values of 0.786 (TUG dataset) and 0.805 (POMA dataset), are thus in line and satisfactory by comparison. Meanwhile, in terms of relevant digital measures, it is notable that not only physical activity and broadly gait-related measures (such as the number of room transitions) but also sleep and rhythmicity measures, such as activity in bed, bed-exit count, or activity fragmentation, were of major importance in discriminating between participants with high and low fall risk (see Fig. 3). Although prospective data would be necessary to form clear conclusions, this may suggest that behavioural data beyond just gait and physical activity may be relevant for fall-risk assessments in older adults. Moreover, results from Piau et al. show that PIR array-based gait speed may help identify future fallers^15^. In this regard, the inclusion of gait-speed information thorough zero-interaction approaches would likely further increase performance on fall-risk related COAs.

In terms of frailty, comparable studies report ROC AUC values between 0.72 and 0.86, based on wearable sensors^51–53^. These results were primarily obtained on the basis of gait and physical activity measures. Additionally, frailty definitions, study type, and participant characteristics differ quite widely, so at best this gives a broad idea of what is possible. With a ROC AUC value of 0.704, our results are on the lower end of this spectrum. However, given the types of sensors we employed, this seems realistic. Indeed, some of the most important measures related to frailty were related to fridge usage, physical activity (room-transition counts), and sleep duration (see Fig. 3), all of which seem plausible as potential digital biomarkers for frailty.

For late-life depression, comparable studies are lacking. Several studies have demonstrated the utility of using wearable-based digital measures in assessing general depression^54–56^. Furthermore, one instance reported on the assessment of late-life depression using PIR-derived information on activities of daily living (ADL)^57^. However, it is unclear whether their methodology prevented data leakage, judging by the unusually high ROC AUC values ≥ 0.95. Our own results, by contrast, show modest performance in assessing late-life depression, with a ROC AUC value of 0.620. While this may be due to the low number of participants with a GDS score above 5 in our cohorts, it could also indicate some inherent difficulties in measuring this outcome. Many of the most important individual measures for late-life depression assessments are related to sleep duration (see Fig. 3), which is known to be associated with depression. More interestingly, variations in fridge usage and behaviour complexity were relevant; however, due to the relatively low discriminative power, further interpretation may not be meaningful.

Regarding the distinction between healthy older adults and those with MCI, recent work has shown ROC AUC values of 0.62–0.80, based on comparable time intervals^20,58^. These values were achieved with wearable devices but also when using additional modalities such as smartphone and computerised assessments. Our result, with a ROC AUC value of 0.780, is thus aligned with similar research and shows that good discriminative performance may potentially be achieved through an entirely passive, zero-interaction set of sensors here as well. Further supporting the plausibility of our results, a respectable body of literature shows how individual digital measures based on PIR and door sensors — such as variability in PIR array-based gait speed^59–61^, ADL regularity^41^, regularity in physical activity^28^, sleep disturbances^62^, and outing duration^63^ — differed between older adults with MCI and healthy controls. Finally, with regards to MCI, highly important measures include those related to physical activity, such as the number of room-transitions or the total amount of PIR-based activity. Moreover, sleep-related measures such as sleep duration, activity in bed, and variation in in-bed activity were found to be important (see Fig. 3). Regarding MCI, however, the most noteworthy finding is the inclusion of various sleep-related heart-rate measures - most importantly, the variation in nightly heart-rate dipping behaviour, where unusually high variation seems indicative of MCI (see Fig. 2). This is especially interesting, as it has not been previously reported in connection with digital measures. However, it is known that heart-rate dipping is associated with cardiovascular disease^64^ and that cardiovascular risk factors may be involved in cognitive decline^65^. As such, it could be beneficial to further investigate the relationship between nightly heart rate and mild cognitive impairment.

Overall, our findings not only suggest that a more comprehensive systems approach towards remote health-monitoring may be promising for long-term clinical care and research, particularly when combined with modern ML approaches, but also demonstrate a potential alternative to commonly employed wearable monitoring of digital measures. As such, although this should be seen as early evidence, employing a digital exhaust, as opposed to using few individual measures, could enable powerful ML derived digital COAs and help to profile and discover novel characteristics of health and disease, eventually empowering the idea of precision (or systems) medicine. The value of a digital exhaust in creating ML derived digital COAs is also supported by the observation that, across all outcomes, the sum of the remaining SHAP values — that is, all digital measures except for the 9 most important ones combined — was highly important in explaining model outcomes (see Fig. 3). Since zero-interaction technologies could be very relevant for long-term remote health-monitoring in older adults, the proposed digital exhaust would also give seniors, that are not comfortable, or not able, to use wearable devices (anymore), a promising alternative. This may be particularly important when considering the still existing digital divide, that was recently shown not to narrow for older adults with serious conditions^66^. Nonetheless, it must be mentioned that most, if not all, digital measures presented could also, at least in theory, be derived by wearable devices, which may be suitable for some but likely not all older adults.

Furthermore, by relying entirely on contactless, zero-interaction technologies, large sets of digital measures can be derived and used without major ethical concerns related to burdening subjects with unnecessary sensing modalities. Best practices laid out by Goldsack et at., for instance, discourage efforts towards sensor-symptoms mapping, which is, to some degree, what a systems approach is doing^67^. However, since in this case sensor technologies respect privacy (no video or audio recordings, for instance) and do not add any additional burden (hence zero-interaction), there are scarcely any downsides, as would occur with adding additional wearables or even active tasks that require interactions. Despite the positives, our findings also suggest that, at least with the presented digital measures and zero-interaction technologies, some modalities may not be easily assessed, such as in our case the assessment of late-life depression. Eventually, we thus believe that the presented digital exhaust has the potential to serve as a baseline set of measures that may be calculated over long time frames (ranging from years to potentially decades), but which could also be supplemented (potentially over shorter time periods) with digital measures based on more specific sensors, such as pillbox sensors, wearables, smartphones, or even non-invasive biomolecular sensors (for instance on the basis of sweat^68^ or saliva^69^), depending on the specific needs, circumstances, and conditions. In clinical care, a baseline set of digital measures could make for a first defense, a basic monitoring layer that helps to indicate when more elaborate, but also more obtrusive and potentially expensive, measurement modalities are necessary (be it based on specific sensing devices, such as a Holter electrocardiogram, or more biological modalities like blood panels or even multi-omics profiling).

Future research should emphasise further analytical and prospective, clinical validation of the included digital measures^70^. Here it will also be of high relevance, to implement clinical grade software infrastructure to support robust long-term collection of the proposed measures as well as integration of new ones. While this will likely require industry participation, a solid first effort has been made with the recently established Collaborative Aging Research Using Technology (CART) initiative, which seeks to make ageing-related digital health approaches more accessible to the broader research community^36^. Moreover, analysing long-term temporal dynamics will be essential, as it would enable the identification of trajectories of certain digital measures or even whole groups thereof. Evaluating trajectories, could be extremely valuable, as was shown by Akl et al., who showcased impressive results regarding MCI classification based on long-term trajectories of several individual digital measures^61^. When considering longitudinal aspects, also concepts around digital resilience biomarkers^69^ may be of interest, by, for instance, monitoring how certain measures change as a result of disturbances to others. For instance, how a night of restless sleep influences certain physiological or activity measures the next day. In addition, future research may seek to combine a digital exhaust, such as the one we utilised, with traditional multi-omics profiling and mobile bio molecule sensing in a deep phenotyping effort. This may enable a wide range of new research insights into ageing and ageing-related conditions, as it adds a new layer of objective information for characterising phenotypes of health and disease. As a final caveat, a digital exhaust such as this should never be assumed to be complete or fixed. Future research will add new digital measures while old measures may be merged if they exhibit closely correlated behaviour. As such, in the immediate future, it may be of major value to add more accurate gait-related digital measures to the introduced set, as these have consistently been shown to be highly important across many ageing-relevant health outcomes. Consequently, they are likely to add significant value. Also the addition of novel contactless sensing technologies that support a zero-interaction approach, could be promising. Good candidates here would be sensors based on radio signal technologies.

Although this work provides promising results we would like to point out some of the major shortcomings. First, some of the introduced measures have not been validated beyond the scope of this research (the use or validation of a digital measure in other studies is indicated in the Supplementary Information). This implies that some measures may not quantify what we hypothesise, which could lead to inaccurate interpretations and conclusions. Here it is also important to stress that associations revealed by the employed model explainability approach do not imply causality. One potential way to overcome this would be to apply approaches around computational causal discovery^71^. Another limitation is the relatively small number of participants used to demonstrate the potential of the presented digital exhaust. Therefore, our results should be treated as early evidence and interpreted with caution, although general tendencies are likely to be valid. Furthermore, in the feasibility demonstration, we use a cross-sectional approach that fails to leverage the temporal trajectories of sequentially collected slices. Indeed, we strongly believe that data collected over multiple years would be necessary to fully explore the utility of digital exhaust based approaches. Here, further longitudinal (ideally over multiple years) studies will be necessary to evaluate this potential. Finally, one drawback of simple PIR and door sensors is that not all digital measures based on this technologies can be calculated when more than one person is living in an apartment. Here a potential solution may be found in wireless radio signal technologies^14,72,73^, that could not only provide more accurate digital measures, compared to simple PIR motion sensors, but should also be able to differentiate between multiple persons, by detecting specific signatures (such as gait characteristics)^74^.

To concluded, we introduce a comprehensive set of digital measures, what may be referred to as a digital exhaust, for long-term remote health-monitoring in the older adult demographic. Overall, the digital exhaust consists of 1268 digital measures derived from 94 hypothesis-driven base measures, covering large parts of a person’s daily activity, behavior and physiology. All included digital measures are derived from a small set of zero-interaction, contactless sensing devices that have been successfully used in numerous ageing-related, long-term, remote-monitoring projects around the world. For each measure, we provide a detailed description, background information, and additional real-world data as supplementary online material (https://narayanschuetz.github.io/digital-exhaust/). While use cases for the introduced digital exhaust are diverse, we demonstrate the case of creating multiple large-scale machine learning-derived digital COAs and evaluate their discriminative performance. To this end, we show how ageing-relevant outcomes such as fall risk, frailty, and MCI may be assessed. Our results with this systems approach not only show that combined information from the digital exhaust significantly outperformed basic demographic information, but also that the digital exhaust based digital COAs could often match the performance reported in studies employing more obtrusive wearable sensors. Finally, we highlight the possibility of using the digital exhaust to discover novel digital biomarker candiates, using a model explainability approach on the basis of the ML models used to create the aforementioned digital COAs. The respective results show that the most important digital measures are reasonable digital biomarker candidates, while also revealing two potentially relevant insights. The first being, that while fall risk may be primarily associated with gait and physical activity, it also potentially exhibits strong associations with sleep-related measures. The second indicating that unusually high variation in nocturnal heart-rate dipping may be uniquely related to MCI.

## Methods

### Creating a zero-interaction digital exhaust

The introduced digital measures are based on three sensor types that have been commonly used in remote-monitoring projects with older adults: PIR sensors, contact door sensors, and a sleep sensor. The PIR motion sensors were placed in the essential rooms of older adults’ apartments. Essential rooms included the living room, bedroom, entrance, bathroom/toilet, and kitchen. The employed PIR sensors sampled with 0.5 Hz, and thus reported activity on or off states every 2 s. The reed switch-based door sensors, meanwhile, were placed at the entrance and the refrigerator door. Both PIR and door sensors were part of the DOMO Care® (DomoHealth SA, Lausanne, Switzerland) home-monitoring system. Finally, for the sleep sensor, we used an EMFIT QS ferroelectret sensor (Emfit Ltd, Vaajakoski, Finland), which was fixed beneath the mattress at approximately chest height. A summary of these three devices, as well as their respective source data streams, is given in Table 3.

**Table 3:** Sensing devices data stream summary.

Sensor	Device	Location	Device type	Data stream	Sampling rate	Channels
PIR Motion	DomoCare	Wall/ Ceiling	Contactless	Activity	0.5 Hz	Entrance
						Toilet
						Bedroom
						Livingroom
						Kitchen
						Others
Door Sensor	DomoCare	Door	Contactless	Open/ Close	Events	Entrance
						Fridge
Bed Sensor	EMFIT QS	Beneath Mattress	Contactless	Heart Rate	0.25 Hz	N/A
				Respiration Rate	0.25 Hz	N/A
				Activity	0.25 Hz	N/A
				Toss & Turns	Events	N/A
				Sleep Phases	0.25 Hz	Awake
						REM
						Deep
						Light
				Raw Lowband	50 Hz	N/A
				Raw Highband	100 Hz	N/A
				Bed Exits	Events	N/A
				Statistics	Daily	N/A

A summary of the contactless pervasive computing devices used, including the respective data streams they provide.

The creation of the digital 94 base measures was mostly hypothesis-driven or based on measures from the existing literature. The majority of base measures was calculated on a daily or nightly basis (for instance, daily total activity, daily outing duration, or average heart rate during a night). Subsequently, we calculated derivates of those measures by means of descriptive statistics over non-overlapping bi-weekly segments, resulting in the final number of 1268 digital measures. While bi-weekly segments may be somewhat arbitrary, two weeks is a sufficient period to capture variation in behaviour that often follows daily or weekly cycles while still being short enough to capture temporally limited behaviours. Additionally, it serves to increase the number of data points (data augmentation) and facilitates the process of working with sensor recordings of various lengths or with data gaps. For cases of certain behavioural or rhythmicity measures, such as Cosinor regression-based measures, raw data from the whole bi-weekly segment was used directly. To avoid the inclusion of measures with insufficient data, we set a minimum number of 10 days for which raw source data was available throughout a given bi-weekly segment; otherwise, the measure was set as missing. The criteria for including a day’s worth of data for each sensor type are explained in detail in the supplementary material (Supplementary Note 1).

**Fig. 4:**
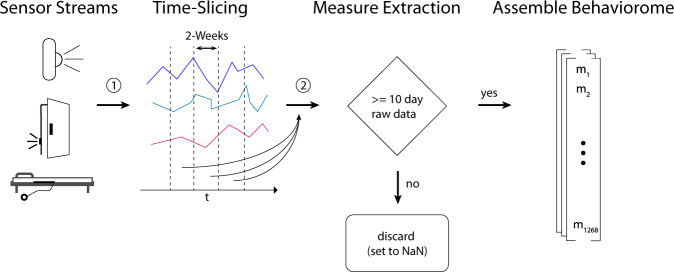
Digital measure extraction flowchart. Shows a broad summary of how digital measures were calculated, starting with raw sensor data from PIR sensors, door sensors, and bed sensors. Raw data streams were first segmented into non-overlapping bi-weekly segments. Then, for each bi-weekly segment, digital measures were calculated. If, for a given measure, less than 10 days of data were present, the measure was encoded as missing, which eventually left 1268 dimensional vectors - one per bi-weekly segment.

For all daily or sub-daily base measures, derivates based on summary statistics were calculated over the bi-weekly segments. Summary statistics include various quantiles, denoted as q*n* (e.g., q10), the interquartile range (iqr), the mean, median ( = q50), coefficient of variation (coefvar), and robust measures of kurtosis and skewness (kr3 and sk3, respectively), following the naming convention proposed by Kim and White^75^. Figure 4 summarises this workflow visually. Eventually, this left 1268 dimensional vectors (one per bi-weekly segment). Of those, 224 dimensions are related to PIR and door sensors, while the remaining 1044 are based on sleep-sensor data. Detailed information regarding the exact calculation of each measure, as well as individual distributions across our cohort, can be found at *online*.

### Example visualisation

We provide an example of averaged digital exhausts with respect to MCI. These visualisations were created by first averaging the digital exhausts on a per-participant basis, followed by z-normalisation. After that, the exhausts were split into the positive and negative outcome groups and averaged once more. Finally, heatmaps for both conditions were created. As a result, the values of individual digital measures > 0 indicate above-average values, while those < 0 indicate below-average values.

### Machine learning based digital clinical outcome assessments

To test the feasibility of using the previously described digital exhaust to create ageing-relevant digital COAs, we used real-world remote-monitoring data from two cohorts of older Swiss adults (pooled age years = 87 ± 7; sex 67% [30/45] female). The original studies were both pilots designed to assess novel computing technologies for ageing-in-place scenarios in the German- and French-speaking cantons of Switzerland^76,77^. They were conducted between 2017 and 2018 and monitored participants over one year with a set of pervasive computing devices and clinical assessments^76,77^. The inclusion criteria between cohorts were similar in the sense that both aimed to recruit a natural sample of community-dwelling older adults (aged ≥ 70 years) who lived alone and without pets. On the other hand, the exclusion criteria between cohorts differed. For cohort 1, the only exclusion criterion was an unwillingness to comply with the study protocol. But, for cohort 2, the exclusion criteria were as follows: (1) severe cognitive impairment rendering the individual unable to follow study protocol (clock-drawing score ≥ 4); (2) skin problems such as irritations, itching, or serious redness; (3) undergoing dialysis; (4) unwillingness to comply with the study protocol; (5) an inability to understand the study aim; or (6) hospitalisation planned within a short period of time^76^. Both studies were conducted based on principles declared in the Declaration of Helsinki and approved by the Ethics Committees of the cantons of Bern and Vaud (KEK-ID: 2016-00406 and CER-VD ID: 2016-00762, respectively). All subjects signed and returned informed consent forms before participating in the study. Detailed participant characteristics and cohort differences are shown in Table 4. The differences between cohorts were statistically examined on the basis of unpaired, two-sided, two-sample *t*-tests (*α* = 0.05). In every analysis involving participant data, all participants with any available data (depending on sensor data and the availability of clinical assessments) were included; this also applies to participants that dropped out of the studies.

**Table 4:** Participant Characteristics.

Characteristic	Cohort 1 (*n* = 24)	Cohort 2 (*n* = 21)	Pooled (*n* = 45)	Differences (*p*-value)
Age (years)	88 ± 7	86 ± 7	87 ± 7	0.50
Sex female (%)	79	52	67	0.06
TUG	14.0 ± 10.0	15.3 ± 4.8	14.5 ± 8.2	0.58
POMA	21.4 ± 7.4	24.1 ± 3.3	22.5 ± 6.1	0.13
EFS	4.6 ± 3.6	5.0 ± 1.7	4.7 ± 2.9	0.65
GDS	2.3 ± 2.3	4.2 ± 3.2	3.1 ± 2.9	0.04
MoCA	20.5 ± 5.1	20.6 ± 3.7	20.5 ± 4.7	0.95

Details characteristics of the participants from the two pilot studies we used in the presented real-world evaluations.

Participants in both cohorts were subject to an overlapping set of standardised clinical assessments. These include the following six assessments: (1) the Timed Up and Go Test (TUG), which is often used in geriatrics to assess fall risk^78^; (2) the Tinneti Performance-Oriented Mobility Assessment (POMA), which, as with the TUG, also measures balance and gait characteristics that are often indicators for elevated fall risk among older adults^79^; (3) the Edmonton Frail Scale (EFS), a frequently used measure of frailty among older adults^80^; (4) the short version (15-item) of the Geriatric Depression Scale (GDS), a commonly used late-life depression screening tool^81^; (5) the Montreal Cognitive Assessment (MoCA), which measures cognitive function and is often used as a brief screening tool for the detection of MCI in older adults^82^. In each cohort, these assessments were planned to be conducted at least once during the one-year study duration. Detailed assessment intervals are summarised in Table 5.

**Table 5:** Clinical assessments and employed cut-off points.

Assessment	Interval cohort 1	Interval cohort 2	Cut-off points	Outcome
TUG	6 weeks	6 weeks	≥12 s^88^	Fall-risk
POMA	half year	half year	<19 points^89^	Fall-risk
EFS	begin/end	begin/end	>5 points^90^	Frailty
GDS	begin/end	begin/end	≥5 points^91^	Depression
MoCA	begin/end	end	<23 points^92^	MCI

Displays an overview of clinical assessments used to evaluate the health status of study participants, including the measurement interval and cut-off points used to divide participants into groups with positive/neutral or negative health outcomes.

To evaluate the potential for creating digital COAs that may help differentiate between positive and negative ageing-relevant health outcomes based on the proposed digital exhaust, we categorise participants into one of the two categories for each clinical assessment. This was done on the basis of validated cut-offs for each assessment, as described before. The respective cut-off values for the negative groups are shown in Table 5. Next, we calculated the digital exhaust for all participants. For each assessment, we then combined the positive/negative labels with the bi-weekly segments of a given participant. If multiple records of the same clinical assessments were obtained throughout the study, we assigned the target label corresponding to the assessment closest in time. After this procedure, we obtained one dataset per assessment.

**Fig. 5:**
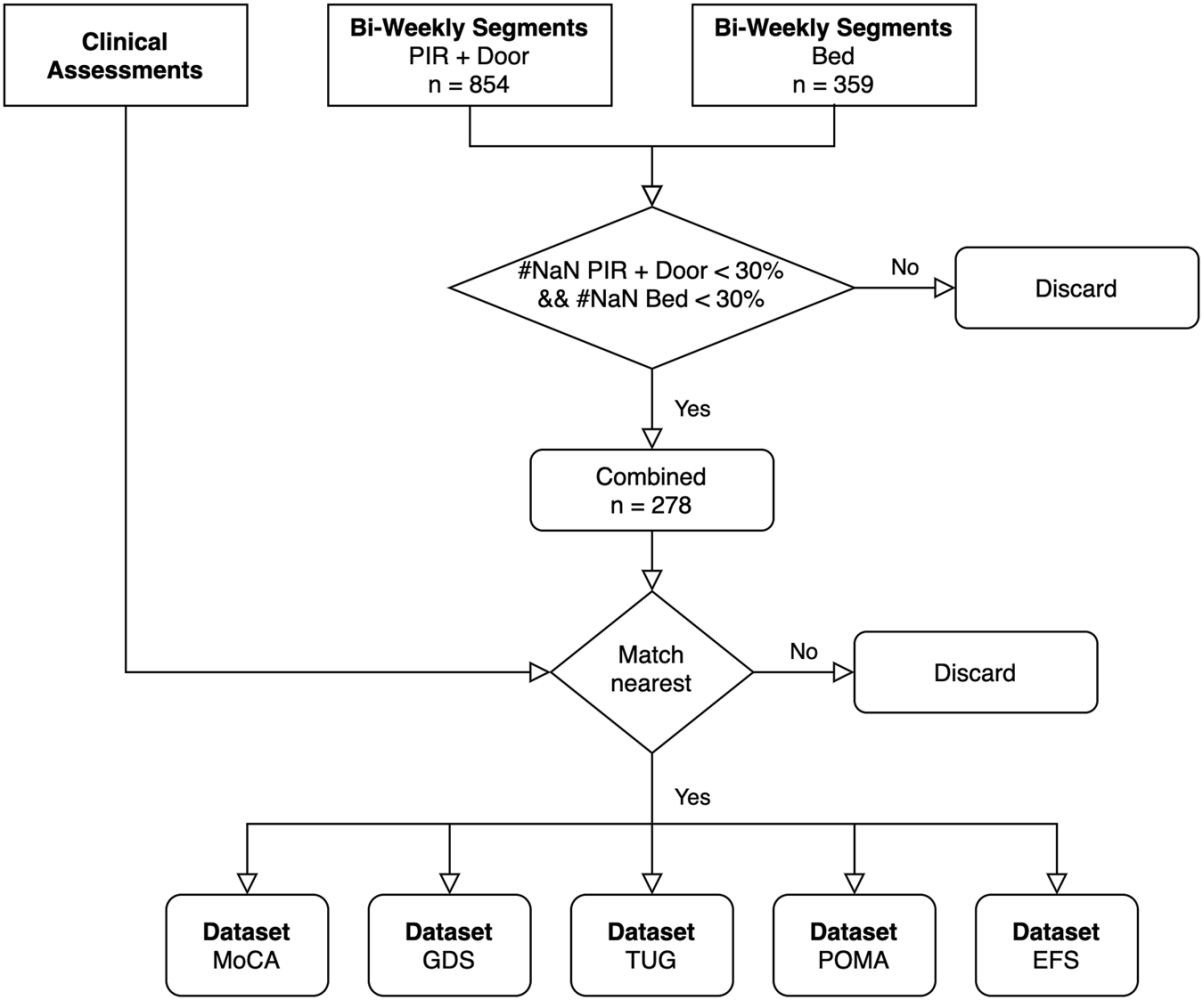
Dataset creation overview. Highlights the workflow of creating datasets, subsequently used for the creation of digital clinical outcome assessments. First, digital measures were separately calculated for the PIR + door and bed sensors and segmented into non-overlapping, bi-weekly segments. After that, the measures from bi-weekly segments, where the percentage of missing digital measures from either sensor system was < 30%, were combined. Next, the clinical assessments from each participant were matched with the respective bi-weekly digital measure vectors to combine 5 datasets --- one for each assessment.

Note that measures derived from PIR and door sensors stem from one sensor system (meaning that technical failure usually affect both sensor types, except for instances were an individual sensor unit failed, which happened rarely), while sleep stems from another sensor; thus, for a bi-weekly segment to be valid, at least 30% of measures from both sensor systems must be valid. This led to a significant reduction in the number of bi-weekly segments, as a large number of sleep sensor data were missing due to technical issues, as has been discussed in prior work^39^. These two issues — lacking sensor data from both PIR/door and bed sensors and the unavailability of respective assessments — are responsible for the generally lower numbers of participants who could be included in this analysis (the exact numbers with regards to each assessment are given in Table 6). In Fig. 5, we present the high-level flowchart of dataset creation.

**Table 6:** Dataset Characteristics.

Dataset	Participants total	Participants with negative outcome	Number Bi-weekly segments
TUG	28	14	277
POMA	28	10	277
EFS	28	10	277
GDS	28	7	277
MoCA	25	16	260

Displayed are the detailed characteristics of the individual outcome datasets. This includes the total number of participants per dataset, the number of participants exhibiting a negative health outcome, as well as the total number of resulting bi-weekly segments (data samples).

A small forenote aimed at a more technically oriented audience: what we call digital measures throughout this work can be seen as synonymous to the more abstract and general term “features”. To evaluate digital COAs based on the digital exhaust, we largely followed the approach set out by Chen et al., albeit with some minor changes^20^. As such, we use the gradient boosting-based XGBoost algorithm^83^ as a classifier, since it generally performs impressively on tabular data, tends to deal reasonably well with high-dimensional feature spaces (even in *p* > > *n*-type scenarios, as here), and can inherently deal with missing values, all of which means it is close to being the gold standard for this kind of application^20,54,84^. Furthermore, gradient boosting-based tree approaches tend to be more easily explainable than modern neural network approaches such as convolutional neural networks, while also retaining high accuracy, especially on tabular data structures^49^. To better account for stochasticity in participant selection, we further adapted the simulation strategy of Chen et al.^20^, in which 70% of participants were repeatedly drawn from the entire participant pool to form a training set, while the remaining 30% were used as a test set (the splits are stratified for the respective clinical assessment labels). This procedure was repeated for 100 iterations. Note, that this way each new draw represents a shuffling of the dataset without introducing data leakage between training and test splits. Throughout each iteration, hyperparameters were first optimised within the training split by means of stratified 3-fold cross-validation coupled with random search (consisting of 50 search trials). For more detailed explanation of this strategy, we refer to the original article by Chen et al. where it is demonstrated in detail^20^.

Eventually, for each iteration, we calculated the Area Under the Receiver-Operating Curve (ROC AUC) and the Area Under the Precision Recall Curve (PrAUC) on the test set, where multiple bi-weekly segments from a single participant were combined into one score by averaging their predictions (soft voting), as was done in^20^. Likewise, if multiple assessment results were available, they were first averaged, which should have also reduced some of the inherent noise; these results were then dichotomised on the basis of the previously introduced cut-off points (see Table 5) to yield a single label per participant. We removed three digital measures (Measure IDs: *iqr_entrance_door_tod_first*, *q50_entrance_door_tod_first*, and *q50_fridge_door_tod_middle*) from the full set for this portion of the analysis, as they were biased towards identifying one of the two cohorts (to account for further less-obvious biases in this regard, we included cohort information in the demographics). Note that the PrAUC is sensitive to label distribution, which means it only lends itself to comparisons within the same dataset. For each assessment, we ran three different scenarios, one with only demographic information (age, sex, and cohort membership) as baseline, one with only the digital exhaust, and one with both the exhaust and demographic information combined. Differences between these scenarios were deemed statistically significant if the 95% CIs of two conditions do not overlap. Model hyperparameter ranges are given in Supplementary Table 1. We used the original Python (version 3.6) implementation of XGBoost (version 1.3.3). Model training was performed on UBELIX (http://www.id.unibe.ch/hpc), the HPC cluster at the University of Bern.

### Discovering novel digital biomarkers

To better understand the role of individual digital measures in machine learning-based COAs, we used SHapley Additive exPlanations (SHAP), a game-theoretic approach for explaining complex machine learning models. With this approach, exact solutions can be found in the case of tree-based models^48,49^. SHAP values have been used fairly extensively in recent biomedical applications^20,85–87^. For each of the assessments, we provide overall global SHAP values across all 100 simulations. That is, we give the mean absolute value of the SHAP values for a given digital measure *m* in a single simulation, summed up over all simulations, as depicted in equation (1).

1 $$SHA{P}_{m}^{global}=\mathop{\sum }\limits_{i=1}^{100}mean(| SHA{P}_{m}^{i}| ).$$

While global SHAP values reveal the overall importance of a given digital measure, they do not say anything about the direction in which the digital measure influences a model. Therefore, we additionally calculate beeswarm plots of the SHAP values. These are based on a model trained over the entire respective dataset, with manually set hyperparameters (reported in the supplementary material). SHAP values were calculated using Python (version 3.6) with the *shap* package (version 0.39.0).

### Reporting summary

Further information on research design is available in the Nature Research Reporting Summary linked to this article.

## Supplementary information

Supplementary Information

Reporting Summary Checklist

Dataset 1

## Data Availability

Original data used in this manuscript may be obtained upon request but will require ethical approval from the responsible authorities. Limited aggregated data are available online (https://narayanschuetz.github.io/digital-exhaust/).

## Code availability

Code used to derive the respective digital measures may be obtained upon request but will not be useful without the exact same sensor setup. A detailed rigorous description is therefore provided on GitHub and in the upplementary Information, including mathematical depictions and pseudo-code (where applicable), such that it is easily possible to replicate each digital measure (https://narayanschuetz.github.io/digital-exhaust/).

## References

[1] Bloom DE, Canning D, Lubet A (2015). Global population aging: Facts, challenges, solutions & perspectives. Daedalus.

[2] Morley JE, Vellas B (2017). Patient-centered (p4) medicine and the older person. J. American Med. Directors Asso..

[3] Au R, Ritchie M, Hardy S, Ang TFA, Lin H (2019). Aging well: Using precision to drive down costs and increase health quality. Adv Geriatric Med. Res..

[4] Hood L (2013). Systems biology and p4 medicine: past. Rambam Maimonides Med. J..

[5] Flores M, Glusman G, Brogaard K, Price ND, Hood L (2013). P4 medicine: How systems medicine will transform the healthcare sector and society. Personalized Med..

[6] Hood L (2017). P4 medicine and scientific wellness: catalyzing a revolution in 21st century medicine. Molecular Front. J..

[7] Gibbs WW (2014). Medicine gets up close and personal. Nature.

[8] Maron JL, Jones GB (2018). How sensors, devices, and biomarkers can transform precision medicine: Perspectives from a clinical and translational science institute. Clinical Therapeutics.

[9] Rantz MJ (2015). A new paradigm of technology-enabled ’vital signs’ for early detection of health change for older adults. Gerontology.

[10] Al-Khafajiy M (2019). Remote health monitoring of elderly through wearable sensors. Multimedia Tools Applications.

[11] Lyons BE (2015). Pervasive computing technologies to continuously assess alzheimer’s disease progression and intervention efficacy. Front. Aging Neurosci..

[12] Jacobson NC, Weingarden H, Wilhelm S (2019). Digital biomarkers of mood disorders and symptom change. NPJ Digital Med..

[13] Piau A, Wild K, Mattek N, Kaye J (2019). Current state of digital biomarker technologies for real-life, home-based monitoring of cognitive function for mild cognitive impairment to mild alzheimer disease and implications for clinical care: Systematic review. J. Medical Internet Res..

[14] Kabelac Z (2019). Passive monitoring at home: A pilot study in Parkinson's disease. Digital Biomarkers.

[15] Piau A (2020). When will my patient fall? sensor-based in-home walking speed identifies future falls in older adults. J. Gerontol.: Series A.

[16] Zhang H, Deng K, Li H, Albin RL, Guan Y (2020). Deep learning identifies digital biomarkers for self-reported parkinson’s disease. Patterns.

[17] Evers LJ (2020). Real-life gait performance as a digital biomarker for motor fluctuations: The parkinson@ home validation study. J. Medical Internet Res..

[18] Coravos A (2019). Digital medicine: A primer on measurement. Digital Biomarkers.

[19] Zhan A (2018). Using smartphones and machine learning to quantify parkinson disease severity: the mobile parkinson disease score. JAMA Neurol..

[20] Chen, R. et al. Developing measures of cognitive impairment in the real world from consumer-grade multimodal sensor streams. In Proceedings of the 25th ACM SIGKDD International Conference on Knowledge Discovery & Data Mining, 2145–2155 (2019).

[21] Vieira, F. G. et al. A machine-learning based objective measure for als disease severity. *Npj Digit Med.* **5**, 45. 10.1038/s41746-022-00588-8 (2022). 10.1038/s41746-022-00588-8 PMC8993812 35396385

[22] Servais, L. et al. Stride velocity 95th centile: Insights into gaining regulatory qualification of the first wearable-derived digital endpoint for use in duchenne muscular dystrophy trials. *J. Neuromuscular Dis.* **9**, 335–346 (2021). 10.3233/JND-210743 PMC9028650 34958044

[23] Kourtis LC, Regele OB, Wright JM, Jones GB (2019). Digital biomarkers for alzheimer’s disease: the mobile/wearable devices opportunity. NPJ Digital Med,.

[24] Dillenseger A (2021). Digital biomarkers in multiple sclerosis. Brain Sci..

[25] Gold M (2018). Digital technologies as biomarkers, clinical outcomes assessment, and recruitment tools in alzheimer’s disease clinical trials. Alzheimer’s & Dementia: Translational Res. Clinical Interventions.

[26] Dorsey ER, Papapetropoulos S, Xiong M, Kieburtz K (2017). The first frontier: digital biomarkers for neurodegenerative disorders. Digital Biomarkers.

[27] Austin J (2016). A smart-home system to unobtrusively and continuously assess loneliness in older adults. IEEE J. Translational Eng. Health Med..

[28] Hayes TL (2008). Unobtrusive assessment of activity patterns associated with mild cognitive impairment. Alzheimer’s & Dementia.

[29] Peek ST (2014). Factors influencing acceptance of technology for aging in place: a systematic review. Int. J. Medical Informatics.

[30] Murphy SL (2009). Review of physical activity measurement using accelerometers in older adults: Considerations for research design and conduct. Preventive Med..

[31] Rogers EM (2001). The digital divide. Convergence.

[32] Cullen, R. Addressing the digital divide. Online information review (2001).

[33] Choi NG, DiNitto DM (2013). The digital divide among low-income homebound older adults: Internet use patterns, ehealth literacy, and attitudes toward computer/internet use. J. Medical Internet Res..

[34] Eruchalu CN (2021). The expanding digital divide: Digital health access inequities during the covid-19 pandemic in new york city. J. Urban Health.

[35] Kaye J (2014). Unobtrusive measurement of daily computer use to detect mild cognitive impairment. Alzheimer’s & Dementia.

[36] Beattie Z (2020). The collaborative aging research using technology initiative: An open, sharable, technology-agnostic platform for the research community. Digital Biomarkers.

[37] Kaye JA (2011). Intelligent systems for assessing aging changes: Home-based, unobtrusive, and continuous assessment of aging. J. Gerontol. Series B: Psychol. Sci. Soc. Sci..

[38] Goonawardene, N., Tan, H.-P. & Tan, L. B. Unobtrusive detection of frailty in older adults. In International Conference on Human Aspects of IT for the Aged Population, 290–302 (Springer, 2018).

[39] Schütz N (2021). Contactless sleep monitoring for early detection of health deteriorations in community-dwelling older adults: Exploratory study. JMIR mHealth uHealth.

[40] Schutz, N. et al. A sensor-driven visit detection system in older adults homes: Towards digital late-life depression marker extraction. *IEEE J. Biomed Health Inform* **26**, 1560–1569 (2021). 10.1109/JBHI.2021.3114595 34550895

[41] Urwyler P (2017). Cognitive impairment categorized in community-dwelling older adults with and without dementia using in-home sensors that recognise activities of daily living. Scientific Rep..

[42] Cook DJ, Schmitter-Edgecombe M (2021). Fusing ambient and mobile sensor features into a behaviorome for predicting clinical health scores. IEEE Access.

[43] Schütz N (2019). Validity of pervasive computing based continuous physical activity assessment in community-dwelling old and oldest-old. Scientific Rep..

[44] Hayes, T. L., Hunt, J. M., Adami, A. & Kaye, J. A. An electronic pillbox for continuous monitoring of medication adherence. In 2006 international conference of the IEEE engineering in medicine and biology society, 6400–6403 (IEEE, 2006). 10.1109/IEMBS.2006.260367 PMC2911441 17946369

[45] Rashidisabet, H. et al. A systems biology approach to the digital behaviorome. *Curr. Opin. Sys. Biol.* **20**, 8–16 (2020).

[46] Wright JM, Jones GB (2018). Harnessing the digital exhaust: incorporating wellness into the pharma model. Digital Biomarkers.

[47] Baker M (2013). The’omes puzzle. Nature.

[48] Lundberg, S. M. & Lee, S.-I. A unified approach to interpreting model predictions. In Guyon, I. et al. (eds.) Advances in Neural Information Processing Systems 30, 4765–4774 (Curran Associates, Inc., 2017). http://papers.nips.cc/paper/7062-a-unified-approach-to-interpreting-model-predictions.pdf.

[49] Lundberg SM (2020). From local explanations to global understanding with explainable ai for trees. Nat. Machine Intelligence.

[50] Sun R, Sosnoff JJ (2018). Novel sensing technology in fall risk assessment in older adults: a systematic review. BMC Geriatrics.

[51] Kumar DP (2020). Sensor-based characterization of daily walking: a new paradigm in pre-frailty/frailty assessment. BMC Geriatrics.

[52] Schwenk M (2015). Wearable sensor-based in-home assessment of gait, balance, and physical activity for discrimination of frailty status: Baseline results of the arizona frailty cohort study. Gerontology.

[53] Park C, Mishra R, Golledge J, Najafi B (2021). Digital biomarkers of physical frailty and frailty phenotypes using sensor-based physical activity and machine learning. Sensors.

[54] Makhmutova, M. et al. Prediction of self-reported depression scores using person-generated health data from a virtual 1-year mental health observational study. In Proceedings of the 2021 Workshop on Future of Digital Biomarkers, 4–11 (2021).

[55] Meyerhoff J (2021). Evaluation of changes in depression, anxiety, and social anxiety using smartphone sensor features: Longitudinal cohort study. J. Medical Internet Res..

[56] Vahia IV, Sewell DD (2016). Late-life depression: A role for accelerometer technology in diagnosis and management. American J. Psychiatry.

[57] Kim J-Y, Liu N, Tan H-X, Chu C-H (2017). Unobtrusive monitoring to detect depression for elderly with chronic illnesses. IEEE Sensors J..

[58] Li, J. et al. Tatc: predicting alzheimer’s disease with actigraphy data. In Proceedings of the 24th ACM SIGKDD International Conference on Knowledge Discovery & Data Mining, 509–518 (2018).

[59] Hayes, T. L., Hagler, S., Austin, D., Kaye, J. & Pavel, M. Unobtrusive assessment of walking speed in the home using inexpensive pir sensors. In 2009 Annual International Conference of the IEEE Engineering in Medicine and Biology Society, 7248–7251 (IEEE, 2009). 10.1109/IEMBS.2009.5334746 PMC2846826 19965096

[60] Dodge H, Mattek N, Austin D, Hayes T, Kaye J (2012). In-home walking speeds and variability trajectories associated with mild cognitive impairment. Neurology.

[61] Akl A, Taati B, Mihailidis A (2015). Autonomous unobtrusive detection of mild cognitive impairment in older adults. IEEE Transac. Biomedical Eng..

[62] Hayes TL, Riley T, Mattek N, Pavel M, Kaye JA (2014). Sleep habits in mild cognitive impairment. Alzheimer Dis. Asso. Disorders.

[63] Petersen J, Austin D, Mattek N, Kaye J (2015). Time out-of-home and cognitive, physical, and emotional wellbeing of older adults: A longitudinal mixed effects model. PloS One.

[64] Eguchi K (2009). Nocturnal non-dipping of heart rate predicts cardiovascular events in hypertensive patients. J. Hypertens..

[65] Ciobica A, Padurariu M, Bild W, Stefanescu C (2011). Cardiovascular risk factors as potential markers for mild cognitive impairment and alzheimer’s disease. Psychiatria Danubina.

[66] Frydman JL, Gelfman LP, Goldstein NE, Kelley AS, Ankuda CK (2022). The digital divide: do older adults with serious illness access telemedicine?. J. Gen. Internal Med..

[67] Goldsack, J. C. & Clay, I. It takes a village: Development of digital measures for computer scientists. In Proceedings of the 2021 Workshop on Future of Digital Biomarkers, 38–44 (2021).

[68] Brasier N, Eckstein J (2019). Sweat as a source of next-generation digital biomarkers. Digital Biomarkers.

[69] van den Brink W (2021). Digital resilience biomarkers for personalized health maintenance and disease prevention. Front. Digital Health.

[70] Goldsack JC (2020). Verification, analytical validation, and clinical validation (v3): the foundation of determining fit-for-purpose for biometric monitoring technologies (biomets). NPJ Digital Med..

[71] Eberhardt F (2017). Introduction to the foundations of causal discovery. Int. J. Data Sci. Analytics.

[72] Su BY, Ho K, Rantz MJ, Skubic M (2014). Doppler radar fall activity detection using the wavelet transform. IEEE Transac. Biomedical Eng..

[73] Saho K, Shioiri K, Inuzuka K (2020). Accurate person identification based on combined sit-to-stand and stand-to-sit movements measured using doppler radars. IEEE Sensors J..

[74] Vandersmissen B (2018). Indoor person identification using a low-power fmcw radar. IEEE Transac. Geosci. Remote Sensing.

[75] Kim T-H, White H (2004). On more robust estimation of skewness and kurtosis. Finance Res. Lett..

[76] Pais B (2020). Evaluation of 1-year in-home monitoring technology by home-dwelling older adults, family caregivers, and nurses. Front. Public Health.

[77] Saner H (2020). Potential of ambient sensor systems for early detection of health problems in older adults. Front. Cardiovascular Med..

[78] Podsiadlo D, Richardson S (1991). The timed “up & go”: A test of basic functional mobility for frail elderly persons. J. American Geriatrics Soc..

[79] Tinetti ME, Williams TF, Mayewski R (1986). Fall risk index for elderly patients based on number of chronic disabilities. American J. Med..

[80] Rolfson DB, Majumdar SR, Tsuyuki RT, Tahir A, Rockwood K (2006). Validity and reliability of the edmonton frail scale. Age Ageing.

[81] Lesher EL, Berryhill JS (1994). Validation of the geriatric depression scale-short form among inpatients. J. Clinical Psychol..

[82] Nasreddine ZS (2005). The montreal cognitive assessment, moca: A brief screening tool for mild cognitive impairment. J. American Geriatrics Soc..

[83] Chen, T. & Guestrin, C. XGBoost: A scalable tree boosting system. In Proceedings of the 22nd ACM SIGKDD International Conference on Knowledge Discovery and Data Mining, KDD ’16, 785–794 (ACM, New York, NY, USA, 2016). 10.1145/2939672.2939785.

[84] Cook, D. Digitally mapping the human behaviorome. In 2020 IEEE International Conference on Pervasive Computing and Communications (PerCom), 1-1 (IEEE, 2020).

[85] Lundberg SM (2018). Explainable machine-learning predictions for the prevention of hypoxaemia during surgery. Nat. Biomedical Eng..

[86] Johnsen PV, Riemer-Sørensen S, DeWan AT, Cahill ME, Langaas M (2021). A new method for exploring gene–gene and gene–environment interactions in gwas with tree ensemble methods and shap values. BMC Bioinformatics.

[87] Li R (2020). Machine learning–based interpretation and visualization of nonlinear interactions in prostate cancer survival. JCO Clinical Cancer Informatics.

[88] Bischoff HA (2003). Identifying a cut-off point for normal mobility: a comparison of the timed ‘up and go’ test in community-dwelling and institutionalised elderly women. Age Ageing.

[89] Faber MJ, Bosscher RJ, van Wieringen PCW (2006). Clinimetric properties of the performance-oriented mobility assessment. Phys. Ther..

[90] Perna S (2017). Performance of Edmonton Frail Scale on frailty assessment: its association with multi-dimensional geriatric conditions assessed with specific screening tools. BMC Geriatr..

[91] da Costa Dias FL (2017). Accuracy of the 15-item Geriatric Depression Scale (GDS-15) in a community-dwelling oldest-old sample: the Pietà Study. Trends Psychiatry Psychother..

[92] Carson N, Leach L, Murphy K (2018). A re-examination of Montreal Cognitive Assessment (MoCA) cutoff scores. Int. J. Geriatr. Psychiatry.

