# Supplementary material for: A systems approach towards remote health-monitoring in older adults: Introducing a zero-interaction digital exhaust
Source: NPJ Digit Med. 2022 Aug 16;5:116. doi: 10.1038/s41746-022-00657-y (PMC9381599; doi:10.1038/s41746-022-00657-y)
Supplement: Supplementary file 2 — Reporting Summary Checklist [file 41746_2022_657_MOESM2_ESM.pdf]

nature portfolio

Corresponding author(s): Narayan Schütz, PhD
Murtenstrasse 50
3008 Bern
Switzerland

Last updated by author(s): May 6, 2022

Reporting Summary

Nature Portfolio wishes to improve the reproducibility of the work that we publish. This form provides structure for consistency and transparency in reporting. For further information on Nature Portfolio policies, see our Editorial Policies and the Editorial Policy Checklist.

Statistics

For all statistical analyses, confirm that the following items are present in the figure legend, table legend, main text, or Methods section.

n/a	Confirmed	
☐	☒	The exact sample size (n) for each experimental group/condition, given as a discrete number and unit of measurement
☐	☒	A statement on whether measurements were taken from distinct samples or whether the same sample was measured repeatedly
☒	☐	The statistical test(s) used AND whether they are one- or two-sided<br>*Only common tests should be described solely by name; describe more complex techniques in the Methods section.*
☒	☐	A description of all covariates tested
☒	☐	A description of any assumptions or corrections, such as tests of normality and adjustment for multiple comparisons
☐	☒	A full description of the statistical parameters including central tendency (e.g. means) or other basic estimates (e.g. regression coefficient) AND variation (e.g. standard deviation) or associated estimates of uncertainty (e.g. confidence intervals)
☒	☐	For null hypothesis testing, the test statistic (e.g. F, t, r) with confidence intervals, effect sizes, degrees of freedom and P value noted<br>*Give P values as exact values whenever suitable.*
☒	☐	For Bayesian analysis, information on the choice of priors and Markov chain Monte Carlo settings
☒	☐	For hierarchical and complex designs, identification of the appropriate level for tests and full reporting of outcomes
☒	☐	Estimates of effect sizes (e.g. Cohen's d, Pearson's r), indicating how they were calculated

Our web collection on statistics for biologists contains articles on many of the points above.

Software and code

Policy information about availability of computer code

Data collection	The following describes data collection tools used in the two pilot studies from which we use data in this article:<br>Emfit Ltd. cloud infrastructure was used to collect the bed sensor data.<br>DomoHealth SA cloud infrastructure was used to collect PIR motion and door sensor data.<br>Pryv SA cloud infrastructure was used to collect clinical assessments and health reports.<br>All data was eventually aggregated in an OmniSci (now HEAVY.AI) analytics database hosted locally at the University of Bern after initial collection.
Data analysis	The shown machine learning derived digital clinical outcome assessment models were trained using Python (version 3.6) implementation of XGBoost (version 1.3.3).<br>Model training was performed on UBELIX (\url{http://www.id.unibe.ch/hpc}), the HPC cluster at the University of Bern.<br>All custom code to run the analysis was implemented using Python 3.6.<br>SHAP values were calculated using Python (version 3.6) with the "shap" package (version 0.39.0).<br>Custom code to run the exact experiment (on our infrastructure) can be obtained upon request but all relevant parts of the work were performed with open source algorithms and the procedure was largely reported in a previous technical journal (Developing measures of cognitive impairment in the real world from consumer-grade multimodal sensor streams).<br>In the supplementary material and online interface we additionally provide each presented digital measure is described in detail, including enough mathematical rigor and pseudo code to be replicated easily.

For manuscripts utilizing custom algorithms or software that are central to the research but not yet described in published literature, software must be made available to editors and reviewers. We strongly encourage code deposition in a community repository (e.g. GitHub). See the Nature Portfolio guidelines for submitting code & software for further information.

Data

Policy information about availability of data

All manuscripts must include a data availability statement. This statement should provide the following information, where applicable:

- Accession codes, unique identifiers, or web links for publicly available datasets
- A description of any restrictions on data availability
- For clinical datasets or third party data, please ensure that the statement adheres to our policy

Original data used in this manuscript may be obtained upon request but will require ethical approval from the responsible authorities.
Limited aggregated data are available online https://narayanschuetz.github.io/digital-behaviorome/.

Field-specific reporting

Please select the one below that is the best fit for your research. If you are not sure, read the appropriate sections before making your selection.

☒ Life sciences ☐ Behavioural & social sciences ☐ Ecological, evolutionary & environmental sciences

For a reference copy of the document with all sections, see nature.com/documents/nr-reporting-summary-flat.pdf

Life sciences study design

All studies must disclose on these points even when the disclosure is negative.

Sample size	Where we used human data, we used retrospective data from two previously reported pilot studies, which we clearly reference in the methods section.
Data exclusions	All participants from the used restrospective data, for which we obtained any data were included, as reported in the manuscript.
Replication	This article is more in the technical realm, thus no classic biological data. So replication as in life science experiments does not really apply. However, to account for randomness in our machine learning models, we used a bootstrapping approach, whereby each model training run was simulated 100 times. We report mean and 95 CI values of those runs.
Randomization	Not relevant to this study as retrospective data from other studies were used and those were observational pilots studies without randomization or intervention.
Blinding	Not relevant to this study as retrospective data from other studies was used and those were observational pilots studies without randomization, intervention, or blinding.

Reporting for specific materials, systems and methods

We require information from authors about some types of materials, experimental systems and methods used in many studies. Here, indicate whether each material, system or method listed is relevant to your study. If you are not sure if a list item applies to your research, read the appropriate section before selecting a response.

Materials & experimental systems

n/a	Involved in the study	
☒	☐	Antibodies
☒	☐	Eukaryotic cell lines
☒	☐	Palaeontology and archaeology
☒	☐	Animals and other organisms
☐	☒	Human research participants
☒	☐	Clinical data
☒	☐	Dual use research of concern

Methods

n/a	Involved in the study	
☒	☐	ChIP-seq
☒	☐	Flow cytometry
☒	☐	MRI-based neuroimaging

Human research participants

Policy information about studies involving human research participants

Population characteristics	Participant characteristics related to the retrospective pilot studies from which we used data are present in the article's methods section:<br>The original studies were both pilots designed to assess novel computing technologies for ageing-in-place scenarios in the German- and French-speaking cantons of Switzerland.<br>They were conducted between 2017 and 2018 and monitored participants over one year with a set of pervasive computing

devices and clinical assessments.
The inclusion criteria between cohorts were similar in the sense that both aimed to recruit a natural sample of community-dwelling older adults (aged >= 70 years) who lived alone and without pets.
On the other hand, the exclusion criteria between cohorts differed. For cohort 1, the only exclusion criterion was an unwillingness to comply with the study protocol.
But, for cohort 2, the exclusion criteria were as follows: (1) severe cognitive impairment rendering the individual unable to follow study protocol (clock-drawing score >= 4); (2) skin problems such as irritations, itching, or serious redness; (3) undergoing dialysis; (4) unwillingness to comply with the study protocol; (5) an inability to understand the study aim; or (6) hospitalisation planned within a short period of time.
Both studies were conducted based on principles declared in the Declaration of Helsinki and approved by the Ethics Committees of the cantons of Bern and Vaud (KEK-ID: 2016-00406 and CER-VD ID: 2016-00762, respectively).

Cohort 1: Age Mean=88 SD=7; Cohort 2: Age Mean=86 SD=7
Cohort 1: Sex female 79%; Cohort 2: Sex female 52%

More detailed participant characteristics can be found in the original publications of the respective studies:
Cohort 1: https://www.frontiersin.org/articles/10.3389/fcvm.2020.00110/full; Cohort 2: https://www.frontiersin.org/articles/10.3389/fpubh.2020.518957/full.

Recruitment

Below it is described how human participants were recruitment in the two pilot studies, from which we used data in this article:
Older adults meeting inclusion criteria were approached by their caregivers regarding study participation.
One study focused on the German speaking part of Switzerland, the other on the French speaking part. Bias could have been introduced as only those older adults willing to participate in such studies would enroll. As a result, conclusions drawn may not apply to the whole population of Swiss older adults but rather to the subset that would enroll in such studies to begin with (this is a bias present in all related research, as it would not be ethical to force people to participate in remote health monitoring studies).

Ethics oversight

The Ethics Committees of the cantons of Bern and Vaud (KEK-ID: 2016-00406 and CER-VD ID: 2016-00762, respectively)

Note that full information on the approval of the study protocol must also be provided in the manuscript.
